# *In silico*, *in vitro,* and *ex vivo* studies of the toxicological and pharmacological properties of the flavonoid 5,7-dihydroxy-3,8,4'-trimethoxy

**DOI:** 10.1590/1414-431X2021e11203

**Published:** 2021-08-13

**Authors:** A.P. Sousa, M.S. Oliveira, D.A. Fernandes, M.D.L. Ferreira, L.V. Cordeiro, M.F.V. Souza, L.M.D. Fernandes, H.D.S. Souza, A.A. Oliveira, H.L.F. Pessoa, R.C.S. Sá

**Affiliations:** 1 Departamento de Fisiologia e Patologia, Centro de Ciências da Saúde, Universidade Federal da Paraíba, João Pessoa, PB, Brasil; 2 Departamento de Ciência Farmacêutica, Centro de Ciências da Saúde, Universidade Federal da Paraíba, João Pessoa, PB, Brasil; 3 Departamento de Química, Centro de Ciências Exatas e Naturais, Universidade Federal da Paraíba, João Pessoa, PB, Brasil; 4 Centro de Ciências Biológicas e da Saúde, Universidade Federal de Campina Grande, Patos, PB, Brasil

**Keywords:** Pavonia glazioviana, Genotoxicity, Hemolytic, Toxicity, Bioavailability

## Abstract

Phytochemical studies of the species *Pavonia glazioviana* were performed. Quercetin, kaempferol, acacetin, and trimethoxylated flavonoid compounds (which present biological activity) were isolated. We aimed to evaluate the *in silico*, *in vitro*, and *ex vivo* toxicity of flavonoid 5,7-dihydroxy-3,8,4'-trimethoxy (Pg-1) obtained from *P*. *glazioviana* through chemical structure analyses, toxicity assessment, and predictive bioactive properties, using human samples in *in vitro* tests. *In silico* analysis suggested that Pg-1 presents a good absorption index for penetrating biological membranes (for oral bioavailability), while also suggesting potential antimutagenic, anticarcinogenic, antioxidant, antineoplastic, anti-inflammatory, anti-hemorrhagic, and apoptosis agonist bioactivities. Assessment of hemolytic and genotoxic effects revealed low hemolysis rates in red blood cells with no cellular toxicity in oral mucosa cells. The reduced cytotoxic activity suggested the safety of the concentrations used (500-1000 µg/mL), and demonstrated the varied interactions of Pg-1 with the analyzed cells. The data obtained in the present study suggested potential therapeutic application, and the non-toxic profile indicated viability for future studies.

## Introduction

The genus *Pavonia cav* (Malvaceae) presents roughly 270 species distributed worldwide. In Brazil, there are 142 registered species, among which 92 are regionally endemic to Northeastern and Southeastern Brazil (1). One example in Northeastern Brazil is *Pavonia glazioviana*, a plant popularly known as ‘*malva-da-chapada*’ or ‘*tampa-cabaça*’ (2). Studies carried out by Oliveira (3) show the presence of flavonoids among the main constituents of the species including quercetin, kaempferol, acacetin, tiliroside, and trimethoxyflavone, which display antibacterial, antifungal, and antioxidant activities (4).

5,7-dihydroxy-3,8,4'-trimethoxy (Pg-1) is a trimethoxylated flavonoid present in *P. glazioviana* for which larvicidal activity has been reported (3), but its bioactive potential and toxicological profile remain unclear. However, it is necessary to analyze the safety levels of new natural molecules with therapeutic potential. Based on this, this study aimed to perform *in silico* tests to determine the theoretical molecular toxicity (chemical structure, toxicity assessment predictions, and its predictive bioactive properties) of Pg-1, isolated from *P. glazioviana*. *In vitro* and *ex vivo* tests with human cells were also carried out to investigate hemolysis and genotoxicity indexes in erythrocytes and oral mucosa cells, respectively.

## Material and Methods

### Plant material

Aerial parts of *P. glazioviana* were collected in February 2015 in Jeremoabo (Bahia, Brazil) at 09°44'34.6"S and 38°52'20.4"W and identified by Prof. Adilva de Souza Conceição (Bahia State University - UNEB). A voucher specimen (registration number 28709) was deposited in the Bahia State University Herbarium (Paulo Afonso Collection). This study was registered at the National System of Genetic Resource Management and Associated Traditional Knowledge (SisGen - A568B8A, Brazil).

### Sample preparation

**Figure 1:**
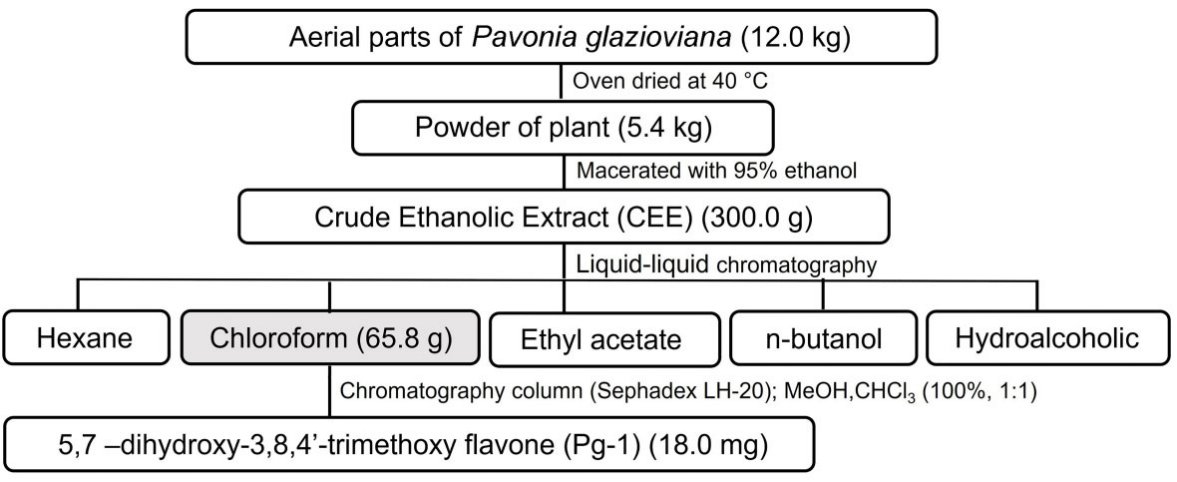
Isolation and purification of flavonoid 5,7-dihydroxy-3,8,4'-trimethoxy (Pg-1) obtained from *P. glazioviana*.

The plant material was oven-dried at 40°C, and subsequently powdered and macerated with 95% ethanol (5 L) for 72 h. The extract solution was dried under reduced pressure at 40°C, yielding crude ethanolic extracts (CEEs), and, after liquid-liquid chromatographic separations using chloroform (CHCl_3_), resulted in their respective fractions. The chloroform fraction (CF) was subjected to Sephadex LH-20 chromatography and spectroscopic procedures (Merck, USA) that allowed the isolation and structural characterization of Pg-1 (Figure 1).

**Figure 2:**
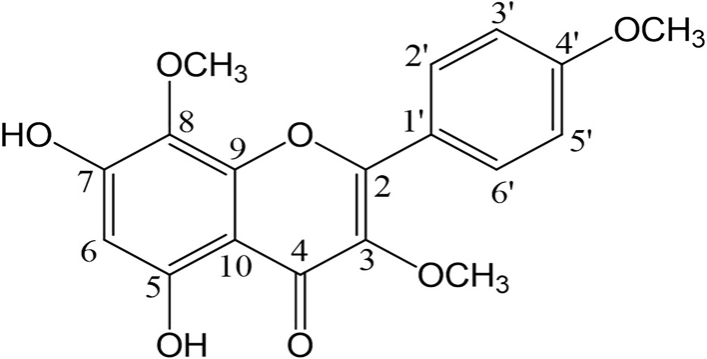
The molecular structure of 5,7-dihydroxy-3,8,4'-trimethoxy (Pg-1).

The Pg-1 molecule (Figure 2) was used in the experiments described in this study. More information on the procedures (Merck) for obtaining the compound and its structural determination are reported in a previous study by Oliveira (3).

### *In silico* analysis

AdmetSAR^®^ software (http://lmmd.ecust.edu.cn/admetsar1/) was used to assess toxicity, and analysis of the following parameters was performed: Gene Inhibition (GI), Ames Toxicity (AT), Potential Carcinogens (PC), Acute Oral Toxicity (AOT), and Carcinogenicity (Car). The software PASS online^®^ (http://www.pharmaexpert.ru/passonline/) was employed to identify the predictive bioactive properties, which predict a compound's activity spectrum as probable activity (Pa) or probable inactivity (Pi). Pa and Pi values can range between 0.000 and 1.000. The compound is considered experimentally active when Pa is greater than Pi (5). Pubchem^®^ (https://pubchem.ncbi.nlm.nih.gov) was used to access the chemical structure of Pg-1.

### Collection of human erythrocytes and oral mucosa cells

The *in vitro* and *ex vivo* assays followed the guidelines provided in the Ethics Code of the World Medical Association and were approved by the Ethics Committee of the Patos University Center (protocol number: 3.621.284). For cell pellet preparation, blood samples (A, B, and O), and buccal mucosa were used from healthy young adults of both sexes between 18 and 40 years of age.

### *In vitro* analysis

For hemolytic activity, red blood cell (RBC) samples were mixed with 0.9% NaCl in a proportion of 1:30 and centrifuged for 5 min (1400 *g*, 28°C) to obtain a white blood cell and platelet-free 0.5% suspension. The tested substance (Pg-1) was added to 2 mL RBC suspensions at the concentrations of 50, 100, 500, and 1000 µg/mL. The control samples were divided into two groups: negative control - RBC suspension (0% hemolysis) and a positive control - RBC suspension with 1% Triton X-100 (100% hemolysis). The material was incubated for 1 h at 22±2°C. Afterwards, the samples were centrifuged for 5 min (1400 *g*, 28°C) and hemolysis was quantified by spectrophotometry at 540 nm wavelength (6).

### *Ex vivo* analysis

For evaluation of the genotoxic effect on oral mucosa cells, cell collection from the oral mucosa of the donors was performed with a cytobrush (smear brush collector) in the cheek area, and the material obtained was placed in 5 mL of 0.9% NaCl (7). Control samples were divided into two groups: cells treated with 0.0005% hydrogen peroxide (positive control) and cells not exposed to treatments (negative control). The cells were washed twice in saline solution and centrifuged for 10 min (900 *g*, 28°C). The cell pellets were re-suspended and kept in 5 mL of saline solution. After the third wash, they were exposed (*ex vivo*) to Pg-1 (50, 100, 500, and 1000 µg/mL) for 30 min; each test was performed in triplicate. The material was centrifuged for 5 min (900 *g*, 28°C) and the supernatant was removed before smear preparation. The cells were homogenized in a vortex and dried on the slides at room temperature for 15 min. Finally, they were fixed with a methanol:acetic acid mixture (3:1) for 15 min (8), and stained with 2% Giemsa. The cells were observed under an optical microscope and about 1000 cells were analyzed per slide. The data are reported as percentages representing the arithmetic average of three measurements (9). The cell toxicity was assessed by the presence of cellular indicators such as micronucleus, bi-nucleation, karyolysis, karyorrhexis, and macronucleus (10).

### Statistical analysis

One-way analysis of variance (ANOVA) and the Bonferroni post-hoc test were used for data analyses, which were performed in GraphPadPrism (version 6.0 for Windows, USA). The experiments were done in triplicate and the results are reported as percentages, representing the arithmetic average of three measurements. Differences were considered statistically significant when P≤0.05.

## Results

### *In silico* analysis

The flavonoid Pg-1 isolated from *P. glazioviana* presented a molar mass of 344.32 g/mol, with 25 atoms. The theoretical physicochemical properties of Pg-1 indicated that the molecule possesses good oral bioavailability according to Lipinski's rule of 5 (Ro5). For good oral bioavailability Pg-1 presents: a molar mass (MM) ≤500 g/mol, number of hydrogen acceptors (nON) ≤10, number of hydrogen bond donors (nOHNH) ≤5, and the number of violations (nviolations) ≤1. The values obtained for Pg-1 were nON: 7, nOHNH: 2, and nviolations: 0, while conforming to the Ro5 criteria (11).

Additional theoretical aspects for bioavailability were analyzed, such as the lipophilicity coefficient (LogP: 3.00), the aqueous solubility coefficient (LogS: -3.52), and the polar topological surface area (TPSA: 98.37 Å2). These suggested that Pg-1 presents a very good absorption index for penetrating biological membranes, in which LogP: ≤5.00, LogS: ≤-4.00, and TSA: ≤140. The molecule classified as soluble with a lipophilic character in accordance with the Ro5 (12), as shown in Table 1.

**Table 1:** Physico-chemical and theoretical aspect of bioavailability.

Parameter	Pg-1
nON	7
nOHNH	2
nviolations	0
LogP	3.00
LogS	-3.52
TPSA	98 Å^2^

nON: number of hydrogen acceptors; nOHNH: number of hydrogen bond donors; nviolations: number of violations; LogP: lipophilicity coefficient; LogS: aqueous solubility coefficient; TPSA: topological polar surface area.

In the toxicity predictions, the ability of the test substance to induce or inhibit mutagenicity was evaluated. Gene Inhibition (GI: 0.825) showed that Pg-1 did not affect gene inhibitors, while AMES Toxicity (AT: 0.925) and Carcinogens (C: 0.918) predicted that Pg-1 had no mutagenic or carcinogenic potential. Pg-1 was classified in category III for Acute Oral Toxicity (AOT: 0.521), which stipulates a mean lethal dose (LD_50_) of greater than 500 mg/kg, and of less than 5000 mg/kg *in vivo* toxicity assays (13). In the present analysis, Carcinogenicity (Car: 0.614) was considered a non-required parameter (Table 2).

**Table 2:** Toxicity *in silico* analysis of Pg-1.

Parameter		
GI	Non-inhibitor	0.825
AT	Non-AMES toxic	0.925
C	Non-carcinogens	0.918
AOT	Category III	0.521
Car	Non-required	0.614

GI: Gene Inhibition; AT: AMES Toxicity; C: Carcinogens; AOT: Acute Oral Toxicity; Car: Carcinogenicity.

The software PASS online^®^ (https://www.ncss.com/software/pass/) was used to perform the predictive analysis of the bioactive properties of Pg-1 (5). The Pg-1 bioactive properties predictions revealed a positive activation probability (Pa) compared to the inactivation probability values (Pi). Additionally, the predictive analyses revealed high probabilities for antimutagenic, anticarcinogenic, antioxidant, antineoplastic, anti-inflammatory, anti-hemorrhagic, and apoptosis agonist activities (Table 3).

**Table 3:** Bioactive properties of Pg-1.

	Pa	Pi
Anti-inflammatory	0.706	0.015
Anti-hemorrhagic	0.528	0.003
Antimutagenic	0.925	0.002
Antineoplastic	0.778	0.015
Antioxidant	0.715	0.004
Anticarcinogenic	0.675	0.010
Apoptosis agonist	0.887	0.005
Apoptosis antagonist	0.211	0.104

Pa: Probability active; Pi: Probability inactive.

### *In vitro* analysis

**Figure 3:**
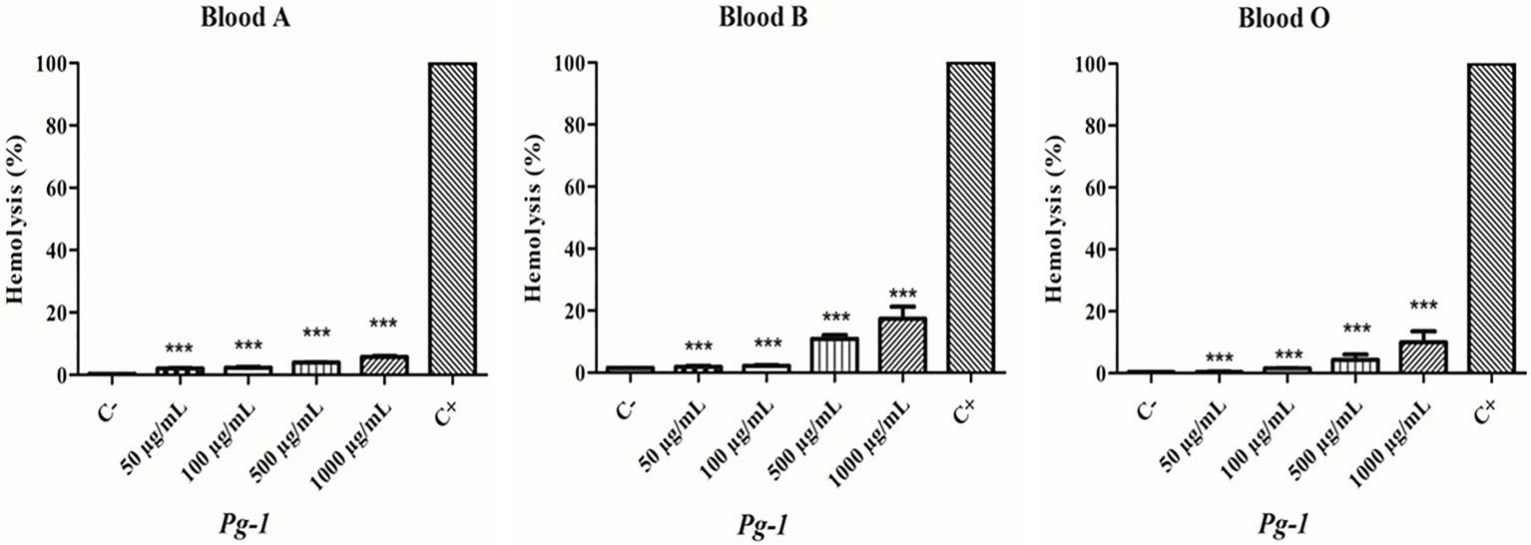
Cytotoxic effect of Pg-1 (*P. glazioviana)* against red blood cells. C-: negative control (erythrocytes 0.5%); C+: positive control (1% Triton X-100). Data are reported as means±SD. ***P<0.001 *vs* positive control (ANOVA). Blood types: A, B, and O.

RBC lysis was assessed and is reported as a percentage (hemolytic potential). RBC lysis is considered low when values are between 0 and 40%, moderate when between 40 and 80%, and high when above 80% (6). After exposure to Pg-1, a low rate of hemolysis was observed at all concentrations tested (50-1000 µg/mL). At 1000 µg/mL, the hemolysis rates were: <17% for type B blood, <9% for type O blood, and <5% for type A blood (Figure 3).

### *Ex vivo* analysis

The parameters used to investigate chromosomal damage after a genotoxic event include: frequency of micronuclei and nuclear abnormalities such as bi-nucleation, karyorrhexis, karyolysis, and macronuclei (10), such as those found in the oral mucosa.

In genotoxicity testing, Pg-1 exhibited low toxicity at all concentrations. In fact, cellular changes were more significant in the groups exposed to the H_2_O_2_ solution (positive control) (Table 4). In the positive control group, the presence of normal cells was <80%. In the negative control group, the presence of normal cells was *>*90%. Pg-1 presented the lowest cell altering function (normal cells >91%) even when used at the highest concentration (1000 µg/mL), yet certain cellular changes were observed (e.g., macronucleus and binucleation), with a lower incidence than that observed for the positive control (H_2_O_2_), and compatible with the results for the negative control, indicating a safe threshold with low toxicity.

**Table 4:** Genotoxic profile of Pg-1.

Group	Micronucleus (%)	Binucleation (%)	Karyolysis (%)	Karyorrhexis (%)	Macronucleus (%)	Normal (%)
Negative control	2.22±0.11	1.88±0.11	0.77±0.22	0.55±0.22	0.001±0.00	94.56±0.22
Positive control	4.00±0.88	4.33±0.38	5.44±0.72	5.11±0.67	4.55±0.80	76.56±3.08
Pg-1						
1000 µg/mL	0.33±0.01*	1.55±0.58*	1.44±0.22*	1.44±0.11*	3.66±0.19*	91.56±0.44*
500 µg/mL	0.22±0.11*	2.33±0.50*	2.55±0.80*	3.66±1.01*	5.33±0.33	85.89±1.78*
100 µg/mL	0.22±0.11*	1.77±0.58*	2.44±0.44*	7.33±0.19	3.55±0.94*	84.67±1.07*
50 µg/mL	0.55±0.11*	3.00±0.33*	2.88±0.11*	1.77±0.58*	6.55±0.58	85.22±0.72*

Data are reported as means±SD. *P<0.05 *vs* positive control (ANOVA).

## Discussion

Flavonoids (abundant in plants) are phenolic compounds whose chemical conformations are related to the antioxidant activities that many plant species have. Its hydroxyl groups capture free radicals from the environment. Phenolic compounds help to reduce oxidative stress and contribute to human health (14).

Studies by Oliveira Filho et al. (15) have suggested that a Pg-1 flavonoid analog, namely 5,7,4'-trimethoxy, also presents good oral bioavailability (nON: 5; nOHNH: 0; LogP: 3.28; MM: 312 g/mol). Thus, these structurally similar flavonoids satisfy the Ro5 and are potential candidates for oral use. Further, the Pg-1 values described for LogS, LogP, and TPSA indicate the potential for intramuscular, cutaneous, and intravenous administration.

The anti-genotoxic effect of Pg-1 is mostly related to its ability to confer cellular protection against oxidative stress. The data obtained in the toxicity parameters, i.e., GI, AT, C, and AOT, suggested that Pg-1 presents a potentially defensive role against free radicals. These results corroborate the findings of Sudha et al. (16), which predict good bioavailability and low mutagenic properties for 5-hydroxy-3',4',7-trimethoxyflavone, a Pg-1 analog. Human use of these bioactive flavonoids would fall well within acceptable toxicity levels.

In different species of the *Pavonia* genus, bioactive compounds have been isolated, such as alkaloids, flavonoids, tannins, and terpenoids. In addition, pharmacological properties and low cytotoxicity have been demonstrated, for example, in the species *P. distinguenda*, which has antibacterial and antineoplastic potential (17), and in *P. varians*, which can be used in the treatment of infections of the digestive system (18).

The bioactive property predictions for Pg-1 corroborate *in silico* analyses of Pg-1 analogs; i.e., 5-hydroxy-3',4',7-trimethoxyflavone and 5,7,4'-trimethoxyflavone, which were previously reported to exhibit biological activities similar to those of Pg-1: being antimutagenic, anticarcinogenic, antioxidant, antineoplastic, and anti-inflammatory (15,16).

Previous studies have reported that flavonoids are the most prevalent class of secondary metabolites in the genus *Pavonia*. The substances usually display low hemolytic activity at <50% (19). The low hemolytic effects of treatment with Pg-1 corroborate the antioxidant and cytoprotective activity of compounds isolated from *P. xanthologea* by Mostardeiro et al. (20). Further, the protective effect of Pg-1 on RBC was demonstrated in the *in silico* bioactive analysis (for anti-hemorrhagic and anti-inflammatory properties), which indicated a higher probability of being active (0.528/0.706) *vs* a probability of being inactive (0.003/0.015).

The genotoxic assay was performed to evaluate if Pg-1 might damage genetic information within oral mucosa cells. DNA damage can disrupt the structure of the genetic material and impair DNA repair mechanisms, resulting in the transformation of normal cells into cancer cells (21).

The results are in agreement with the predictive analysis outcomes, referencing the potential role of Pg-1 in apoptosis induction in view of its probability of being active (0.887) *vs* its probability of being inactive (0.005). This is corroborated by reduced apoptosis inhibitory activity, also considering the probability of being active (0.211) *vs* the probability of being inactive (0.104). *In vitro* and *in silico* approaches have evidenced the antiproliferative and apoptosis-induction activities of flavonoids. The Pg-1 analog 5,7,4'-trimethoxyflavone, for example, has been shown to exert antioxidant-related protection while inhibiting proliferation and inducing cell death in breast cancer cells (22).

The data obtained in the present study suggested that Pg-1 is a safe molecule with potential therapeutic applications. The *in silico* analysis indicated that Pg-1 possesses a good biological membrane absorption index for oral bioavailability. In the *in silico* toxicity test, Pg-1 presented the potential to protect cells against free radicals, in addition to exhibiting potential antimutagenic, anticarcinogenic, antioxidant, antineoplastic, anti-inflammatory, anti-hemorrhagic and apoptosis agonist effects. The *in vitro* and *ex vivo* hemolytic and genotoxic investigations revealed low RBC hemolytic activity and no toxicity in the oral mucosa cells. It is important that more studies are performed, aiming to investigate the toxicological profile of Pg-1 with other human cell lines, since there is a promising bioactivity of this molecule.
